# A novel pan-Nox inhibitor, APX-115, protects kidney injury in streptozotocin-induced diabetic mice: possible role of peroxisomal and mitochondrial biogenesis

**DOI:** 10.18632/oncotarget.18540

**Published:** 2017-06-16

**Authors:** Guideock Kwon, Md Jamal Uddin, Gayoung Lee, Songling Jiang, Ahreum Cho, Jung Hwa Lee, Sae Rom Lee, Yun Soo Bae, Sung Hwan Moon, Soo Jin Lee, Dae Ryong Cha, Hunjoo Ha

**Affiliations:** ^1^ Graduate School of Pharmaceutical Sciences, College of Pharmacy, Ewha Womans University, Seoul, Korea; ^2^ Department of Life Science, Ewha Womans University, Seoul, Korea; ^3^ Aptabio Therapeutics Inc, Yongin-si, Korea; ^4^ Department of Internal Medicine, Division of Nephrology, Korea University, Seoul, Korea

**Keywords:** APX-115, diabetic kidney disease, mitochondria and peroxisome, oxidative stress, pan-Nox inhibitor

## Abstract

NADPH oxidase (Nox)-derived reactive oxygen species (ROS) are increasingly recognized as a key factor in inflammation and extracellular matrix accumulation in diabetic kidney disease. APX-115 (3-phenyl-1-(pyridin-2-yl)-4-propyl-1-5-hydroxypyrazol HCl) is a novel orally active pan-Nox inhibitor. The objective of this study was to compare the protective effect of APX-115 with a renin-angiotensin system inhibitor (losartan), the standard treatment against kidney injury in diabetic patients, on streptozotocin (STZ)-induced diabetic kidney injury. Diabetes was induced by intraperitoneal injection of STZ at 50 mg/kg/day for 5 days in C57BL/6J mice. APX-115 (60 mg/kg/day) or losartan (1.5 mg/kg/day) was administered orally to diabetic mice for 12 weeks. APX-115 effectively prevented kidney injury such as albuminuria, glomerular hypertrophy, tubular injury, podocyte injury, fibrosis, and inflammation as well as oxidative stress in diabetic mice, similar to losartan. In addition, both APX-115 and losartan treatment effectively inhibited mitochondrial and peroxisomal dysfunction associated with lipid accumulation. Our data suggest that APX-115, a pan-Nox inhibitor, may become a novel therapeutic agent against diabetic kidney disease by maintaining peroxisomal and mitochondrial fitness.

## INTRODUCTION

Diabetic kidney disease (DKD) is a key microvascular complication of diabetes and the most common cause of end-stage kidney disease (ESKD) [1]. Oxidative stress is a key pathogenic factor responsible for the initiation and progression of DKD [2–5]. Since clinical trials of several antioxidants against oxidative stress-associated tissue injury have failed [6], inhibiting reactive oxygen species (ROS) generation might become a promising strategy to treat DKD.

Nicotinamide adenine dinucleotide phosphate (NADPH) oxidases (Noxs) are the major sources of ROS in mammalian cells. Noxs also play vital roles in ROS generation in the kidney [7] under high glucose and pro-diabetic conditions [8, 9]. Nox4 is mainly responsible for ROS generation in the kidney [10]. In the experimental models of chronic kidney disease (CKD) including DKD, Nox4 expression and activity are increased in the kidney [11–17]. In a streptozotocin (STZ)-induced type 1 diabetes rodent model, increased expression of Nox4 is associated with ROS-induced kidney damage [15–17], while Nox4 knockout can protect STZ-induced diabetic mice against glomerular injury [18]. Anti-sense oligonucleotides specific for Nox4 can inhibit renal ROS generation, hypertrophy, and matrix expansion in diabetic rats [15]. Nox1/4 dual inhibitor GKT137831 can block high glucose-induced activation of Nox4-associated oxidative stress and pro-fibrotic signaling in mouse proximal tubular epithelial cells [13]. In addition, GKT137831 and GKT136901 can effectively reduce oxidative stress, albuminuria, and kidney fibrosis in mouse models of type 1 and type 2 diabetes [19, 20].

On the other hand, inflammation characterized by macrophage infiltration and activation is involved in the progression of DKD [21]. Nox2 is the major Nox isoform responsible for macrophages activation [22]. Nox2 is expressed also in kidney tissues [23, 24] and tubular epithelial cells [25]. Therefore, targeting all Nox isoforms might be a better strategy against ROS-induced kidney injury in diabetes. In this respect, APX-115 (ewha-18278) has been recently verified as a novel orally active pan-Nox inhibitor that can protect ovariectomy-induced osteoporosis [26] and type 2 diabetes-induced renal injury [27].

Lipid accumulation is associated with oxidative stress and mitochondrial dysfunction in DKD [28]. In addition to mitochondria, peroxisomes have been recently recognized for their important roles in lipid metabolism and ROS homeostasis in mammals. Peroxisome dysfunction in peroxisomal biogenesis disorders of human patients and animal models will disrupt lipid metabolism including fatty acid (FA) oxidation, leading to elevated levels of plasma FA and increased lipid accumulation in the central nervous system, liver, and kidney [29–31]. Peroxisome also participates in ROS homeostasis because they have various oxidases that generate H_2_O_2_ as a reaction by-product and catalase that can metabolize H_2_O_2_ to oxygen and water [32]. Our previous study has demonstrated that peroxisomal dysfunction is associated with oxidative stress in DKD [33]. However, the effect of renotherapeutic agents on peroxisomal fitness has not been studied.

Therefore, the objective of this study was to compare the effect of APX-115 with a renin-angiotensin system inhibitor (losartan), the standard treatment against kidney injury in diabetic patients, on kidney injury in STZ-induced type 1 diabetic mice. With respect to lipid metabolism, we evaluated the effect of both APX-115 and losartan on peroxisomal as well as mitochondrial biogenesis.

## RESULTS

### General characteristics of experimental animals

As expected, STZ-induced diabetic mice showed significantly less body weight gain but higher levels of blood glucose, hemoglobin A1c (HbA1c), urine volume, and kidney weight than controls. Oral administration of APX-115 for 12 weeks did not significantly affect hyperglycemia or any of these parameters in diabetic mice (Table 2). Losartan did not affect hyperglycemia but decreased kidney weight and urine volume in diabetic mice (Table 2).

**Table 1:** Primers used for real time RT-PCR analysis

Gene	Primer sequences
CPT1 (mouse)	Forward 5′- GTGACTGGTGGGAGGAATAC-3′ Reverse 5′- GAGCATCTCCATGGCGTAG-3′
FAS (mouse)	Forward 5′-TGCCTCGGGAATGGAAAG-3′ Reverse 5′-ATGGTAGTCTCCCCATCGTCATA-3′
Fibronectin (mouse)	Forward 5′-TGCCTCGGGAATGGAAAG-3′ Reverse 5′-ATGGTAGTCTCCCCATCGTCATA-3′
KIM1 (mouse)	Forward 5′-ACATATCGTCGAATCACAACGAC-3′ Reverse 5′-ACAAGCAGAAGATGGGCATTG-3′
MCP1 (mouse)	Forward 5′-CTTCTGGGCCTGCTGTTCA-3′ Reverse 5′-CCAGCCTACTCATTGGGATCA-3′
Nox1 (mouse)	Forward 5′-AGCCATTGGATCACAACCTC-3′ Reverse 5′-AGAAGCGAGAGATCCATCCA-3′
Nox2 (mouse)	Forward 5′-TGCACCATGATGAGGAGAAA-3′ Reverse 5′-CCACACAGGAAAACGCCTAT-3′
Nox4 (mouse)	Forward 5′-TGGCCAACGAAGGGGTTAAA-3′ Reverse 5′-GATGAGGCTGCAGTTGAGGT-3′
NGAL (mouse)	Forward 5′-GGCCAGTTCACTCTGGGAAA-3′ Reverse 5′-TGGCGAACTGGTTGTAGTCC-3′
SREBP1c (mouse)	Forward 5′- GGAGCCATGGATTGCACATT-3′ Reverse 5′- AGGAGGGCTTCCAGAGAGGA-3′
NRF1 (mouse)	Forward 5′- AGCACGGAGTGACCCAAAC -3′ Reverse 5′- TGTACGTGGCTACATGGACCT -3′
TFAM (mouse)	Forward 5′- ATTCCGAAGTGTTTTTCCAGCA-3′ Reverse 5′- TCTGAAAGTTTTGCATCTGGGT-3′
TGFβ1 (mouse)	Forward 5′-CTTTAGGAAGGACCTGGGTT-3′, Reverse 5′-CAGGAGCGCACAATCATGTT-3′
TNFα (mouse)	Forward 5′-CGTCAGCCGATTTGCTATCT-3′ Reverse 5′-CGGACTCCGCAAAGTCTAAG- 3′
18S (mouse)	Forward 5′-CGAAAGCATTTGCCAAGAAT-3′ Reverse 5′-AGTCGGCATCGTTTATGGTC-3′

**Table 2:** General characteristics of experimental animals

Parameters	Control	DM	DM+ APX-115	DM+ losartan
Body weight (g)	26.4 ± 0.3	22.4 ± 0.5^*^	22.4 ± 0.4^*^	24.0 ± 0.9^*^
Kidney weight (g)	0.17 ± 0.01	0.21 ± 0.10^*^	0.21 ± 0.01^*^	0.19 ± 0.01^*,†^
Kidney/body weight (mg/g)	7.0 ± 1.0	10.0 ± 1.0^*^	9.5 ± 2.0^*^	8.0 ± 2.0^†^
Blood glucose level (mg/dL)	214 ± 15	506 ± 16^*^	468 ± 21^*^	443 ± 33^*^
HbA1c (%)	4.2 ± 0.1	8.0 ± 0.4^*^	7.5 ± 0.3^*^	7.0 ± 0.4^*^
Urine volume (mL/24 h)	1.0 ± 0.2	17.3 ± 2.4^*^	13.4 ± 2.5^*^	11.0 ± 2.6^*,†^

Diabetes was induced through intraperitoneal injection of STZ (50 mg/kg) for five days. Mice were then treated with APX-115 and losartan for 12 weeks. At 12 weeks after STZ injection, all mice were sacrificed and blood samples were collected for measurement of plasma glucose level. Data are presented as the means ± SE of 10–12 mice/group; *p < 0.05 vs. control, ^†^*p* < 0.05 vs. DM.

### APX-115 prevents albuminuria, hyperfiltration, and glomeruler hypertrophy in STZ-induced diabetic mice

**Figure 1:**
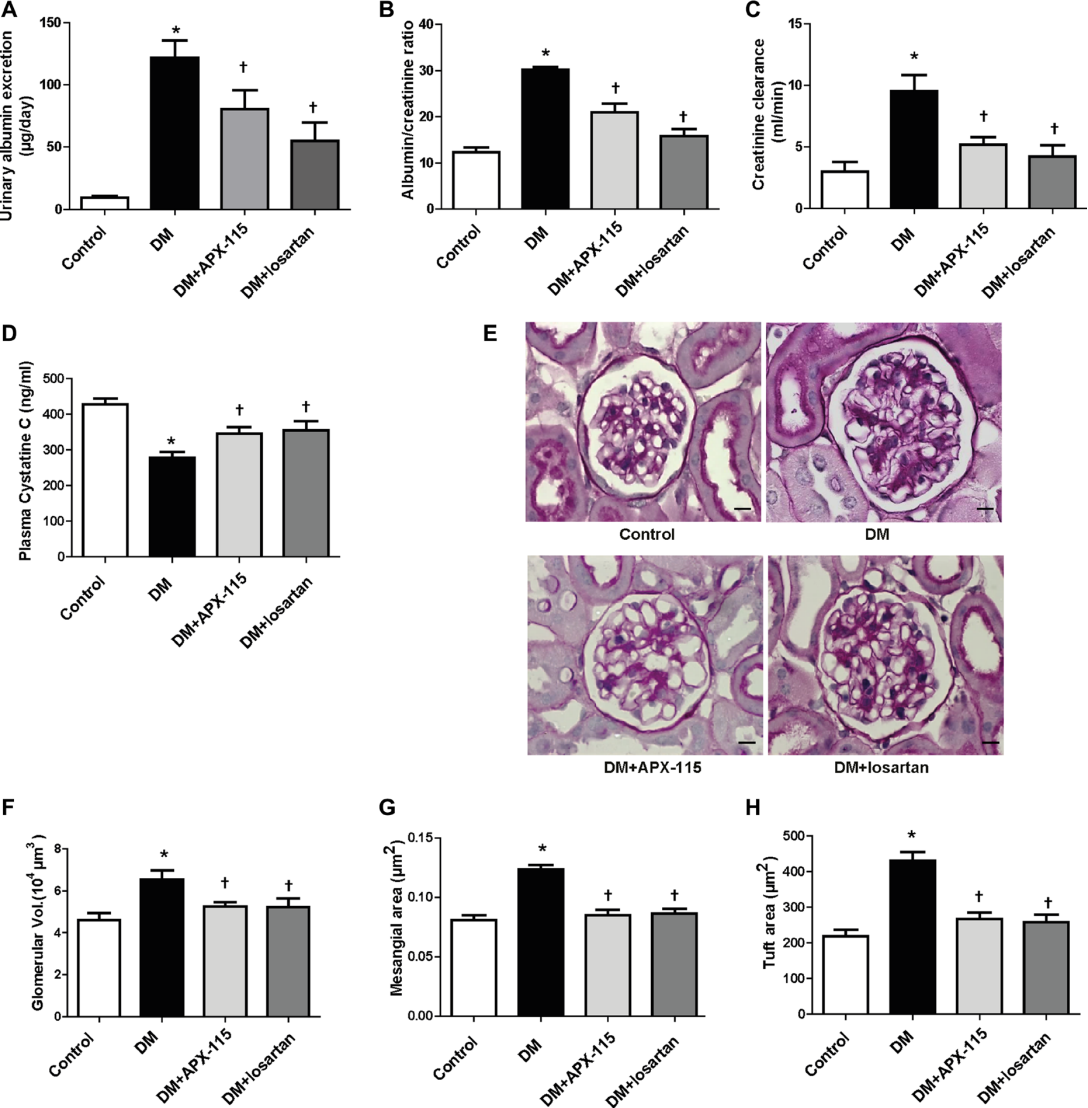
Effect of APX-115 on kidney function and morphology Diabetes was induced in mice by intraperitoneal injection of STZ (50 mg/kg). Then APX-115 (60 mg/kg/day) or losartan (1.5 mg/kg/day) was administered orally for 12 weeks to diabetic mice. After 12 weeks, urine and blood samples were collected for analysis of (**A**) urinary albumin excretion, (**B**) albumin/creatinine ratio, (**C**) creatinine clearance rate, and (**D**) plasma cystatin C. (**E**) Kidneys were fixed in paraffin and cut into 3 μm sections that were subsequently stained with PAS reagent. Scale bar: 10 μm; original magnification: 630×. After PAS staining, (**F**) glomerular volume, (**G**) mesangial area, and (**H**) tuft area were analyzed using Image-Pro Plus 4.5.1. DM, STZ-induced diabetic mice. Data are presented as means ± SE of 10–12 mice/group; *p < 0.05 vs. control, ^†^*p* < 0.05 vs. DM.

APX-115 treatment markedly decreased urinary albumin excretion compared to diabetic controls (Figure 1A and 1B). The antialbumiuric effect of losartan on diabetes was confirmed (Figure 1A and 1B). Diabetic mice showed increased creatinine clearance and decreased plasma cystatin C levels as compared to control, indicating increased glomerular filtration rate. Such hyperfiltration of glomeruli was attenuated by both APX-115 and losartan (Figure 1C and 1D).

Results of periodic acid–schiff (PAS) staining showed that glomerular volume, fractional mesangial area, and tuft area were significantly increased in diabetic mice. These were significantly decreased by APX-115 or losartan (Figure 1E–1H).

### APX-115 inhibits tubular and podocyte injury and kidney fibrosis in STZ-induced diabetic mice

**Figure 2:**
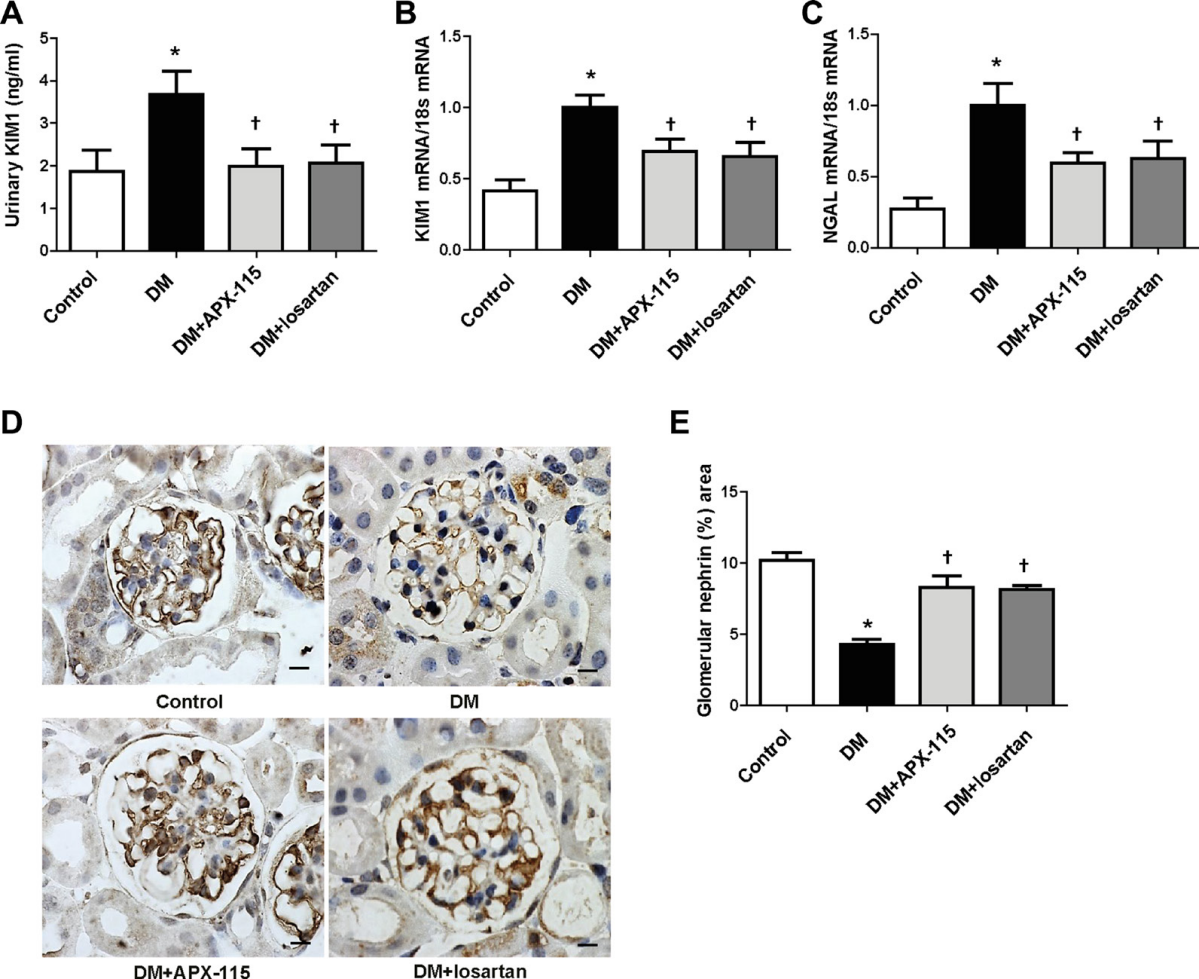
Effect of APX-115 on tubular and podocyte injury in STZ-induced diabetic mice Tubular injury markers such as (**A**) urinary KIM1 by ELISA and (**B**) KIM1 and (**C**) NGAL mRNA levels in kidney tissues using real-time PCR were measured. (**D** and **E**) Paraffin-embedded kidney sections were stained with anti-nephrin antibodies (1:100; Scale bar: 10 μm; original magnification: 630×). Data are presented as means ± SE of 10–12 mice/group; *p < 0.05 vs. control, ^†^*p* < 0.05 vs. DM.

**Figure 3:**
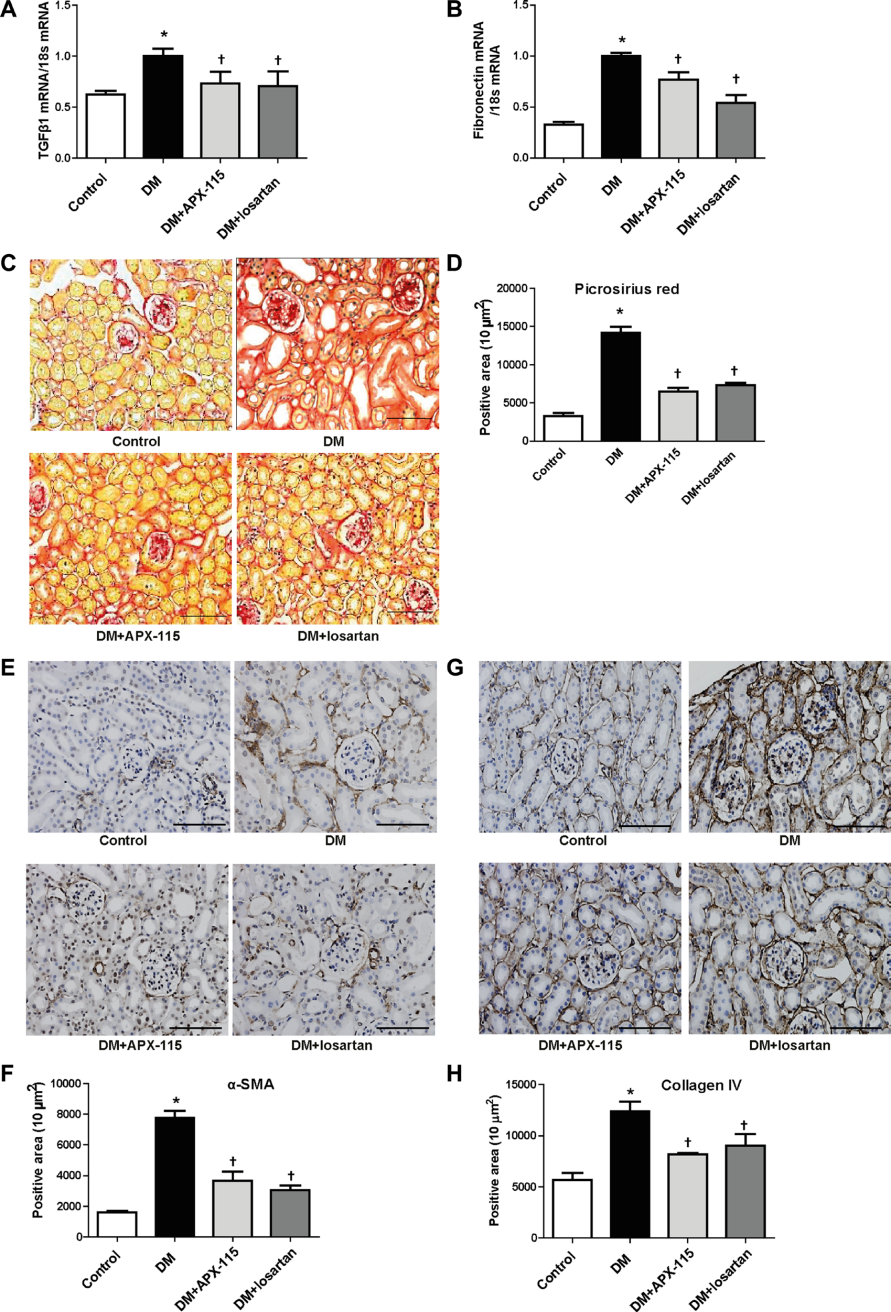
Effect of APX-115 on fibrosis in STZ-induced diabetic mice The mRNA levels of fibrosis markers (**A**) TGFβ1 and (**B**) fibronectin in kidney tissues were measured using real-time PCR. Paraffin-embedded kidney sections were stained with (**C** and **D**) picrosirius red stain, (**E** and **F**) anti-αSMA (1:200), and (**G** and **H**) anti-collagen IV (1:200) antibodies. Original magnification: 200×; scale bar: 100 μm. Data are presented as means ± SE of 5 mice/group; *p < 0.05 vs. control, ^†^*p* < 0.05 vs. DM.

We then examined tubular damage by analyzing the expression of kidney injury molecule1 (KIM1) and neutrophil gelatinase-associated lipocalin (NGAL) levels. STZ-induced diabetic mice showed increased urinary KIM1 as well as kidney KIM1 and NGAL mRNA expression (Figure 2A–2C). They were significantly inhibited by APX-115 or losartan (Figure 2A–2C). Nephrin immunostaining showed that APX-115 or losartan treatment significantly reversed diabetes-induced inhibition of nephrin expression (Figure 2D and 2E), suggesting that APX-115 or losartan could reverse podocyte injury in diabetes. The mRNA expression levels of transforming growth factor β1 (TGFβ1) and fibronectin were increased in the kidneys of diabetic mice. They were significantly inhibited by APX-115 or losartan (Figure 3A and 3B). In line with gene expression of fibrotic markers, picrosirius red staining showed that APX-115 treatment significantly reduced diabetes-induced collagen deposition in the kidney (Figure 3C and 3D). Further, immunostaining showed increased protein expression of α smooth muscle actin (αSMA) (Figure 3E and 3F) and collagen IV (Figure 3G and 3H) in diabetic kidneys, which were inhibited by APX-115 or losartan treatment. These data suggest that APX-115 is a potent inhibitor of tubular/podocyte injury and fibrosis in STZ-induced diabetic kidneys.

### APX-115 inhibits kidney inflammation and oxidative stress in STZ-induced diabetic mice

**Figure 4:**
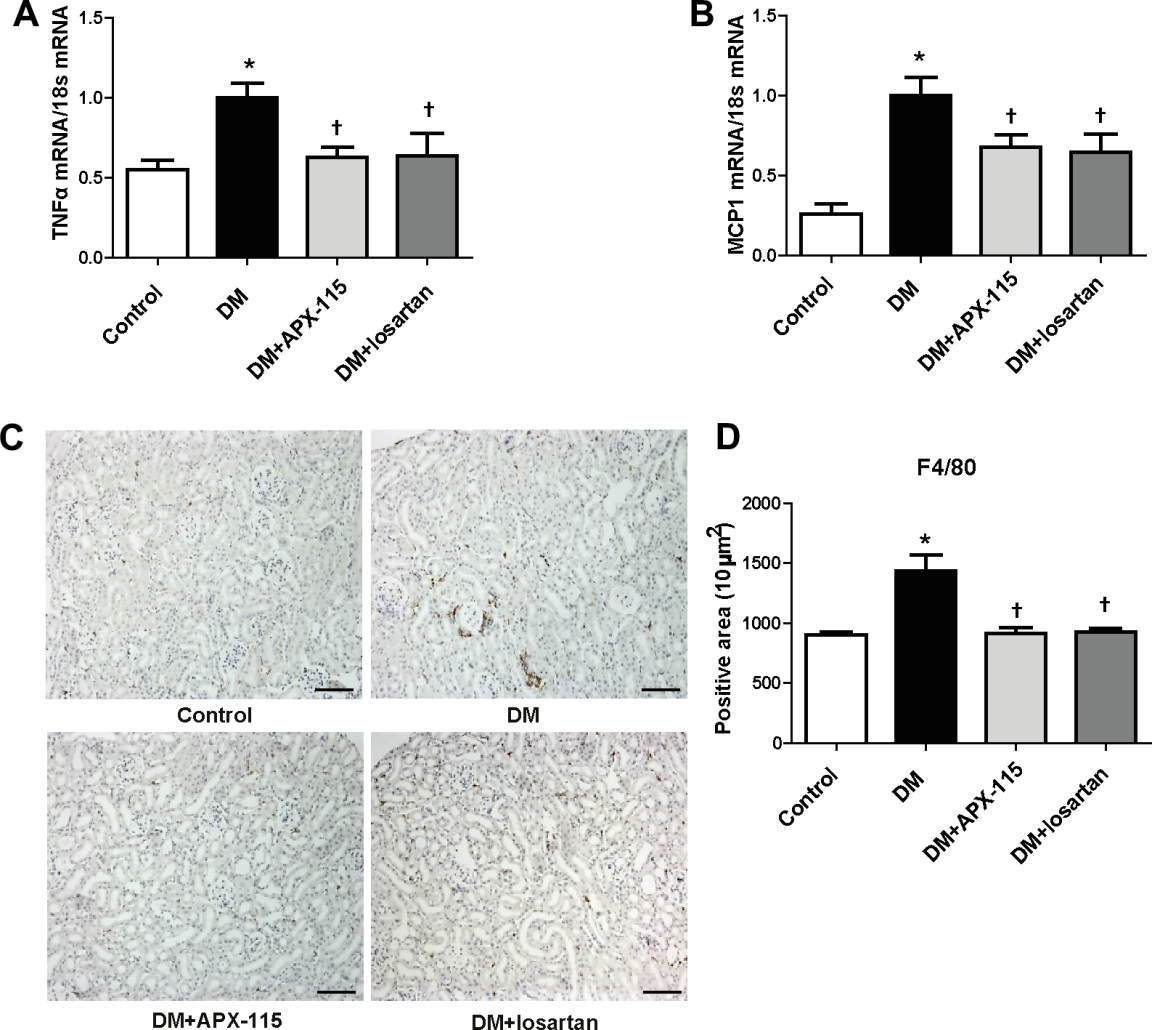
Effect of APX-115 on inflammation in STZ-induced diabetic mice The mRNA levels of inflammation markers (**A**) TNFα and (**B**) MCP1 in kidney tissue were measured using real-time PCR. (**C** and **D**) Paraffin-embedded kidney sections were stained anti-F4/80 antibodies (1:200; original magnification: 100×; scale bar: 100 μm). Data are presented as means ± SE of 5 mice/group; *p < 0.05 vs. control, ^†^*p* < 0.05 vs. DM.

Tumor necrosis factor α (TNFα) and monocyte chemoattractant protein 1 (MCP1) mRNA expression levels were increased in diabetic kidneys. They were significantly reduced by APX-115 or losartan (Figure 4A and 4B). In addition, macrophage infiltration as measured by F4/80 immunostaining was increased in the tubulointerstitium of diabetic mice. Either APX-115 or losartan treatment attenuated macrophage infiltration in the tubulointerstitium of diabetic mice (Figure 4C and 4D).

**Figure 5:**
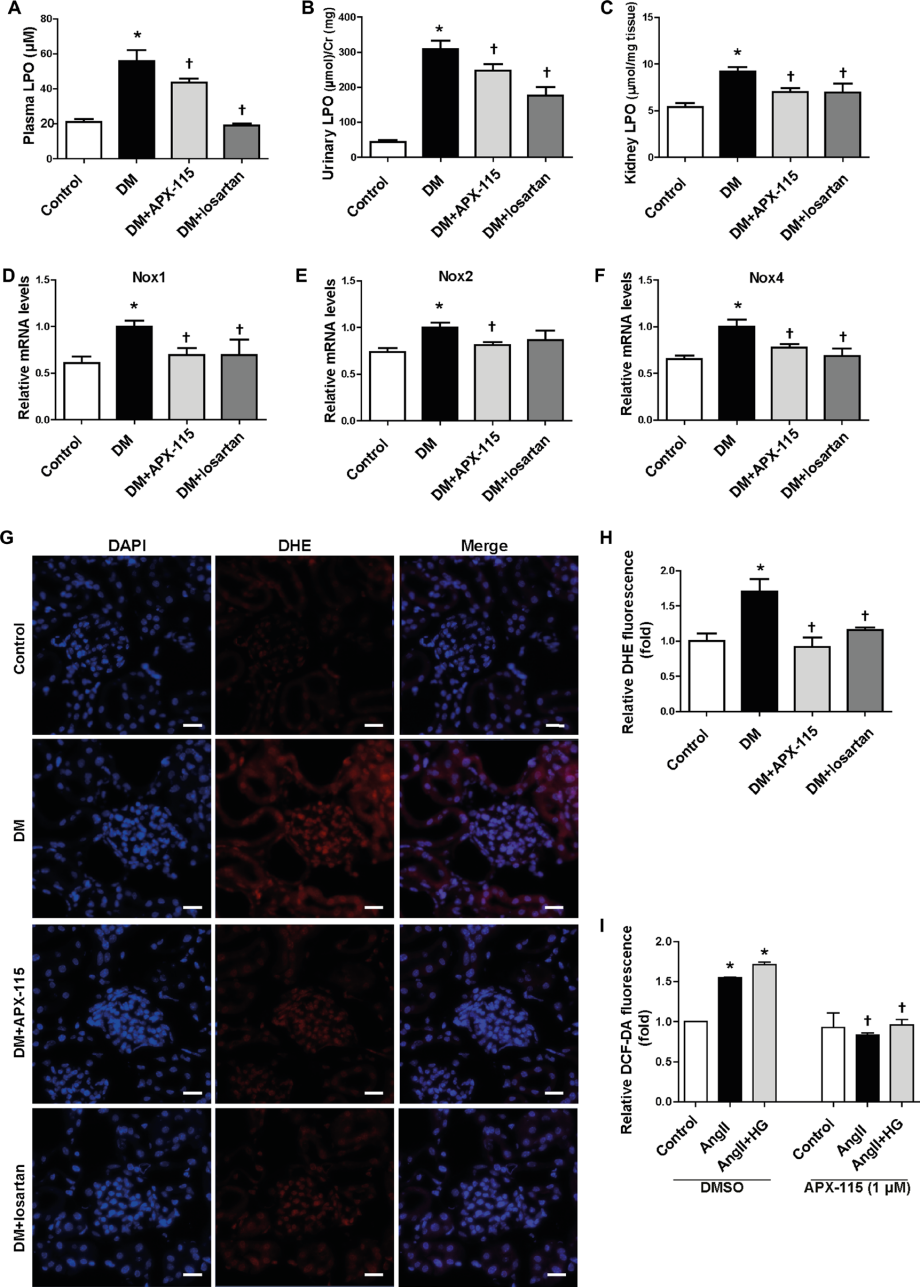
Effect of APX-115 on oxidative stress in STZ-induced diabetic mice (**A**) Plasma LPO, (**B**) urinary LPO, (**C**) kidney tissue LPO, (**D**) Nox1, (**E**) Nox2, and (**F**) Nox4 mRNA expression levels in kidneys were measured using real-time PCR. (**G** and **H**) Frozen kidney sections were stained with DHE at 5 µM (original magnification: 400×; scale bar: 20 μm). (A–H) Data are presented as means ± SE of 10–12 mice/group; *p < 0.05 vs. control, ^†^*p* < 0.05 vs. DM. (**I**) Mesangial cells were incubated with or without APX-115 (1 µM) for 30 min and stimulated with or without 30 mM high glucose (HG) for 24 h followed by angII for 30 min. After that cells were incubated with 10 µM DCF-DA for 10 min and the fluorescence intensity was measured with a Zeiss vision system. Data are presented as means ± SE of at least 2 independent experiments; *p < 0.05 vs. control, ^†^*p* < 0.05 vs. angII or angII+HG in DMSO.

To confirm the anti-oxidative effect of APX-115, we measured plasma, urine, and kidney lipid hydroperoxide (LPO). As expected, APX-115 significantly inhibited diabetes-induced plasma, urine, and kidney LPO (Figure 5A–5C). 8-oxo-dG (Supplementary Figure 1A and 1B) and nitrotyrosine (Supplementary Figure 1C and 1D) accumulation in the kidneys of diabetic mice was significantly reversed by APX-115 or losartan treatment. Nox1, Nox2, and Nox4 mRNA levels were significantly increased in diabetic kidneys. Interestingly, they were significantly inhibited by APX-115 (Figure 5D–5F). Superoxide production as measured by dihydroethidium (DHE) staining was also significantly reversed by APX-115 or losartan (Figure 5G and 5H). To confirm the *in vivo* results, we employed mesangial cell culture system to determine the effects of APX-115 or losartan on angiotensionII (angII) with or without high glucose-induced intracellular ROS by 2′,7′-dichlorofluorescin diacetate (DCF-DA) staining. As expected, ROS generation was inhibited by APX-115 or losartan in mesangial cells (Figure 5I). Taken together, these results suggest that APX-115 may improve diabetes-induced inflammation and oxidative stress, similar to losartan.

### APX-115 inhibits lipid accumulation and regulates mitochondrial and peroxisomal biogenesis in kidney of STZ-induced diabetic mice

**Figure 6:**
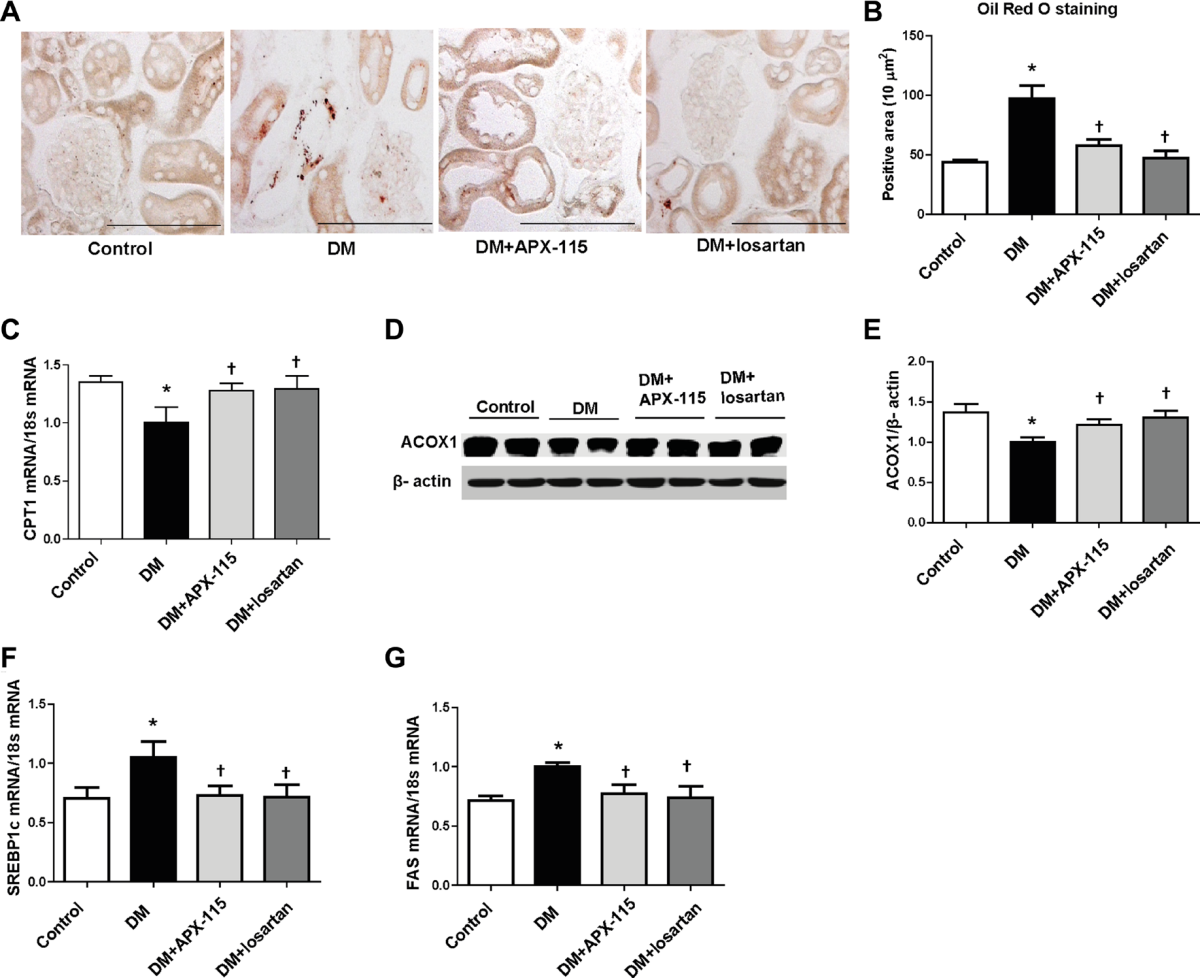
Effect of APX-115 on lipid accumulation in kidney of STZ-induced diabetic mice (**A**) Frozen kidney sections were stained Oil Red O staining (original magnification: 200×; scale bar: 100 μm). (**B**) Quantification of (A). (**C**) mRNA expression of CPT1, (**D** and **E**) protein expression of ACOX1, and (**F** and **G**) mRNA expression of lipogenic markers (SREBP1c and FAS) in kidney tissues. Data are presented as means ± SE of 10–12 mice/group; *p < 0.05 vs. control, ^†^*p* < 0.05 vs. DM.

Oil Red O staining was performed to determine the effect of APX-115 on lipid accumulation. Results showed that diabetes-induced lipid accumulation in the kidney was effectively reduced by APX-115 (Figure 6A and 6B). STZ treatment significantly decreased the expression of lipolytic enzyme such as carnitine palmitoyltransferase 1 (CPT1) mRNA (Figure 6C) and acyl-CoA oxidase 1 (ACOX1) protein (Figure 6D and 6E) but increased mRNA levels of lipogenic enzymes such as sterol regulatory element-binding protein1c (SREBP1c) and fatty acid synthase (FAS) (Figure 6F and 6G) which were reversed by APX-115 or losartan.

**Figure 7:**
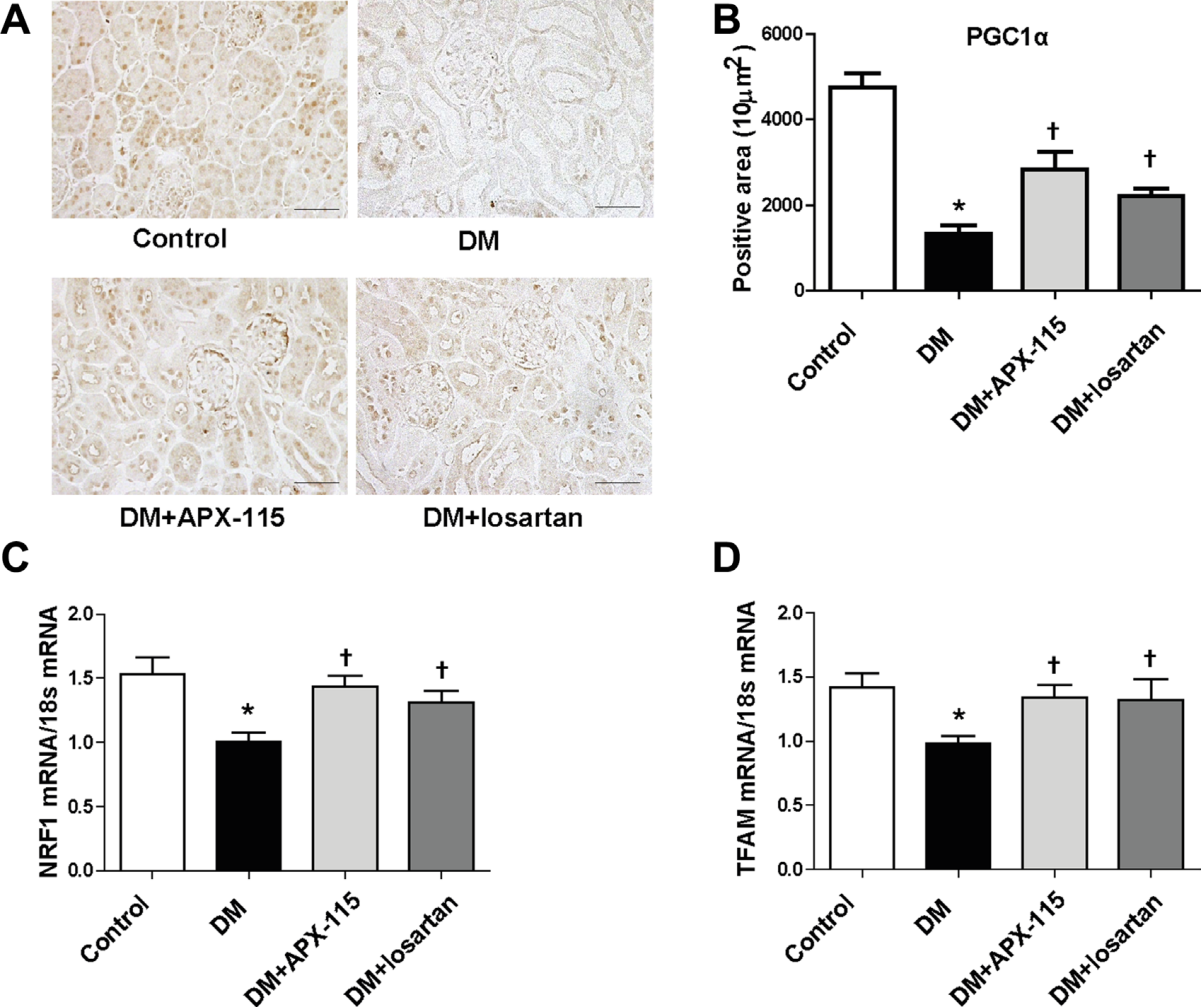
Effect of APX-115 on mitochondrial biogenesis in STZ-induced diabetic mice (**A**) Paraffin-embedded kidney sections were stained with anti-PGC1α antibody (1:200; original magnification: 200×; scale bar: 100 μm). (**B**) Quantification of PGC1α stained positive area. mRNA expression levels of NRF1 (**C**) and TFAM (**D**) in kidney tissues were measured. Data are presented as means ± SE of 10–12 mice/group; *p < 0.05 vs. control, ^†^*p* < 0.05 vs. DM.

Immunostaining of peroxisome proliferator-activated receptor-coactivator1α (PGC1α) showed that APX-115 or losartan significantly restored diabetes-induced suppression of PGC1α in the kidney (Figure 7A and 7B). In addition, mRNA expression levels of nuclear respiratory factor 1 (NRF1) and mitochondrial transcription factor A (TFAM) were significantly restored by APX-115 or losartan in diabetic kidneys (Figure 7C and 7D). These results suggest that APX-115 has potential role in mitochondrial biogenesis of diabetic kidneys.

**Figure 8:**
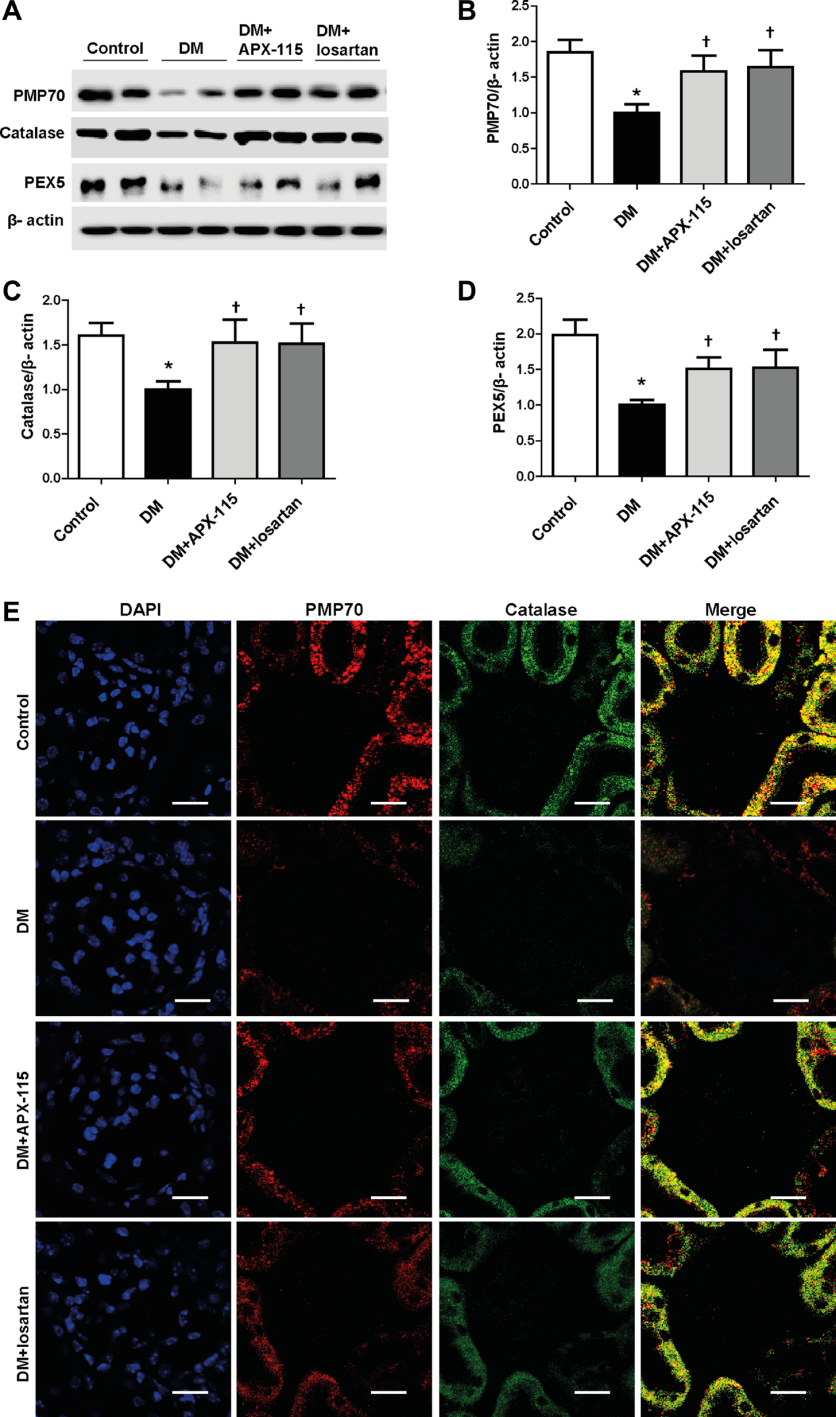
Effect of APX-115 on peroxisomal biogenesis in STZ-induced diabetic mice (**A**) Protein expression levels of PMP70, catalase, and PEX5 in kidneys. (**B**) Quantification of PMP70, (**C**) quantification of catalase, and (**D**) quantification of PEX5. Protein densities were normalized against β-actin. (**E**) Paraffin-embedded kidney sections were stained with anti-PMP70 and anti-catalase antibodies (1:200; original magnification: 600×; scale bar: 20 μm). Data are presented as mean ± SE of 10–12 mice/group; *p < 0.05 vs. control, ^†^*p* < 0.05 vs. DM.

STZ treatment significantly decreased peroxisomal biogenesis based on mRNA expression levels of peroxisomal membrane protein70 (PMP70), catalase, and peroxin5 (PEX5), which was significantly reversed by APX-115 or losartan in diabetic mice (Figure 8A–8D). In order to confirm these results, we employed immunostaining for PMP70 and catalase. Both PMP70 and catalase expressed relatively higher in tubular epithelial cells than glomeruli (Figure 8E). Interestingly, diabetes-induced suppression of PMP70 and catalase were reversed by APX-115 or losartan in the kidneys (Figure 8E), suggesting that APX-115 could also regulate peroxisomal biogenesis in diabetic kidneys.

## DISCUSSION

The present data demonstrated that orally administrated APX-115, a pan-Nox inhibitor, could exert a renoprotective effect in STZ-induced diabetic mice as much as losartan. APX-115 at a dose that could inhibit renal oxidative stress effectively improved tubular and podocyte injury, fibrosis, and inflammation in diabetic kidneys. In addition, APX-115 treatment significantly restored the down-regulation of peroxisomal as well as mitochondrial biogenesis in diabetic kidneys.

Diabetic mice showed increased oxidative stress as measured by LPO, nitrotyrosine, 8-oxo-dG, and DHE. Such oxidative stress was attenuated by treatment with APX-115 or losartan. In addition, intracellular ROS measured by DCF-DA staining in mesangial cells cultured under angII with or without high glucose was also inhibited by APX-115 or losartan. Interestingly, treatment of APX-115, a pan-Nox inhibitor significantly decreased mRNA levels of Nox1, Nox2, and Nox4. These results suggest the positive feedback loop between Nox activity and Nox expression as previously described [34]. Furthermore, at the end of the study, both APX-115 and losartan treatment markedly decreased urinary albumin excretion and creatinine clearance in STZ-induced diabetic mice. Along with remarkable improvement in kidney function, KIM1, NGAL as well as nephrin expression were restored after the administration of APX-115 or losartan. In addition, APX-115 treatment inhibited kidney fibrosis and inflammation markers in STZ-induced diabetic mice. In our previous study, APX-115 treatment suppresses i) high glucose-induced upregulation of NF-κB p65, Nox2, Nox4, MCP1, and profibrotic molecules such as TGFβ1, plasminogen activator inhibitor-1, and collagen IV in cultured podocytes and ii) high glucose-induced ROS and fibronectin production in mesangial cells [27]. Altogether, these findings indicate that APX-115 has renoprotective effects in diabetic mice, similar to losartan.

Consistent with our observation, GKT13783 (inhibitor of Nox1/4) which is currently in phase 2 clinical trials has protective effects against kidney injury in type 1 and type 2 diabetic mice [19, 20]. Although Nox4 is the most important player among Nox isoforms in the kidney, evidence also indicates that Nox2 might also be involved in various kidney injuries including DKD [23, 24, 35, 36]. In addition, APX-115 which inhibits not only Nox1/4 but also Nox2 [26] is more effective than GKT13783 in suppression of some parameters of diabetic kidney injury [27]. Thus, pan-Nox inihibitor, APX-115 might have better effects than a Nox1/4 inhibitor in preventing DKD. Yet, a few questions related to our findings remain to be answered, such as possibility of increased infection and mortality due to long-term Nox2 inhibition, as evident by complete Nox2 deletion [37]. In fact, a previous study has shown that genetic deletion of Nox2 does not protect against the major features of DKD [38]. Additionally, it should be recognized that Nox4 protects against vascular inflammation and oxidative stress [39] as well as kidney disease [40, 41], although the mechanisms involved in those protective effects of Nox4 are not clear. It is also imperative to study the delayed treatment effect of APX-115 and effect of combination therapy with both APX-115 and losartan on DKD.

Consistent with the results of this study, type 1 diabetic mice induced by multiple low-doses of STZ exhibit lipid accumulation in the kidneys [33, 42, 43]. Lipid deposition along with upregulation of SREBP1c and FAS mRNA in diabetic kidneys were significantly reversed by APX-115 or losartan in this study. Lipid accumulation in diabetic kidney is also associated with decreased fatty acid β-oxidation. Treatment with APX-115 or losartan effectively restored CPT1 mRNA and ACOX1 protein expression in diabetic kidneys, suggesting that APX-115 plays a role in fatty acid β-oxidation in both mitochondria and peroxisomes.

Since dysregulated mitochondrial function plays an important role in cellular metabolism, inflammation, and fibrosis [28], we also measured markers of mitochondrial biogenesis. As expected, diabetes-induced down-regulation of NRF1 and TFAM mRNA expression was significantly altered by APX-115 or losartan. This was further supported by the expression of PGC1α, a master regulator of mitochondria biogenesis. In line with our results, it has been reported that oxidative stress-induced mitochondrial dysfunction can be reversed by losartan in type 1 diabetic kidneys of rats [44].

Importantly, diabetic kidneys show peroxisomal dysfunction associated with oxidative stress and lipid accumulation [33]. Suppressed levels of peroxisomal biogenesis markers such as PMP70, catalase, and PEX5 in kidneys of STZ-induced diabetes mice were recovered by APX-115 or losartan. It has been reported that decreased catalase activity can result in metabolic-oxidative imbalance and accelerate the progression of DKD [45]. However, the mechanism of Nox- or angII-induced peroxisomal dysfunction and lipid accumulation in kidney injury also remains to be clarified.

**Figure 9:**
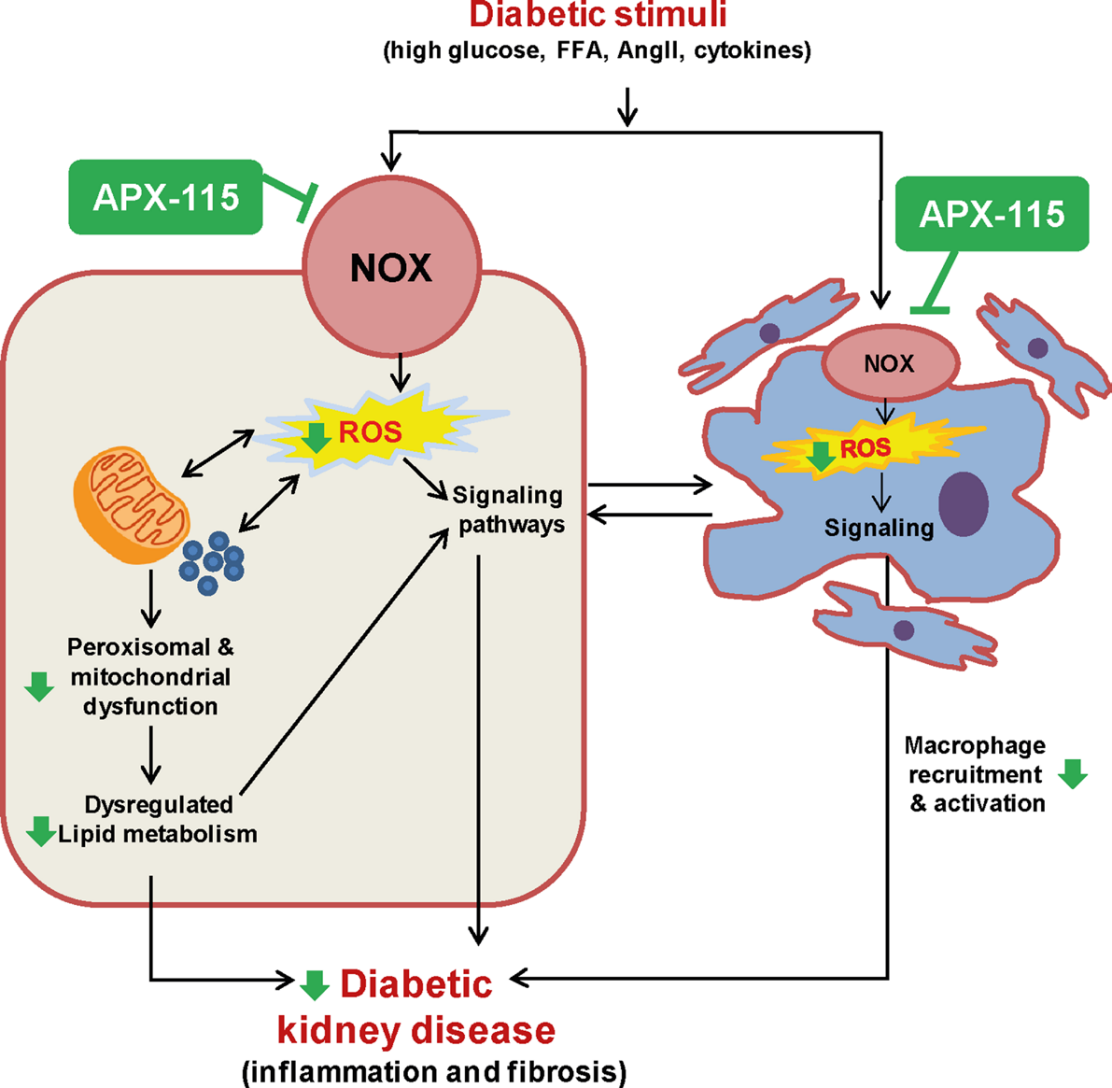
Suggested scheme of therapeutic effects of APX-115 on STZ-induced diabetic kidney injury We summarize here our overall findings schematically, with a focus on how Noxs-mediated ROS production may be a trigger for increased oxidative metabolism leading to mitochondrial and peroxisomal dysfunction associated with lipid accumulation as well as activation of different signaling pathway leading to increased inflammatory responses and profibrotic factors in DKD. In addition, ROS triggers kidney cells to secret cytokines which potentiate recruitment and activation of macrophages and then, the activated macrophages also contribute to DKD. Interestingly, pan-Nox inhibitor, APX-115 attenuates most of the ROS-mediated effects (shown as green arrows), suggesting that APX-115 may become a new therapeutic strategy against DKD.

In conclusion, our findings support that pan-Nox inhibition by APX-115 might have renoprotective potential as much as losartan in DKD. Oxidative stress, inflammation, albuminuria, lipid accumulation, glomerulosclerosis, tubulointerstitial fibrosis, and imbalance in mitochondrial and peroxisomal homeostasis were all elevated in diabetic kidneys which were significantly prevented by APX-115 (schematically presented in Figure 9). These results suggest that APX-115 may become a new therapeutic option in preventing DKD.

## MATERIALS AND METHODS

### Materials

Chemicals and antibodies were obtained from Sigma-Aldrich Company (St. Louis, MO, USA) and Cell Signaling Technology (Danvers, MA, USA), respectively, unless otherwise stated.

### Animals

Six-week-old male C57BL/6 mice (Japan SLC Inc., Hamamatsu, Japan) were used in this study. They were divided into four groups: (i) control group, (ii) diabetic group, (iii) diabetic group treated with APX-115, and (iv) diabetic group treated with losartan. Diabetes was induced by intraperitoneal injection of 50 mg/kg STZ for 5 days. Age-matched control mice were injected with an equivalent volume of sodium citrate buffer (100 mM sodium citrate, 100 mM citric acid, pH 4.5). APX-115 at 60 mg/kg/day or losartan at 1.5 mg/kg/day were orally administered to diabetic mice for 12 weeks. Doses of APX-115 or losartan have been determined based on our preliminary data. Control mice were injected with an equivalent volume of 0.5% methylcellulose, the vehicle used for APX-115. Mice were monitored at least once a day during the experimental period. All mice were sacrificed at 12 weeks after STZ injection via anesthesia with 16.5% urethane (10 mL/kg). All animal experiments were approved by the Institutional Animal Care and Use Committee (IACUC No. 14-051) of Ewha Womans University.

### Measurement of blood parameters

Blood samples were collected with a heparinized syringe before mice were sacrificed. Blood glucose level was determined using the glucose oxidase method. HbA1c level was determined using the DCA2000 HbA1c reagent kit (SIEMENS Healthcare Diagnostics, Inc., Tarrytown, NY, USA). Plasma creatinine level was determined using a Detect X Serum Creatinine Detection Kit (Arbor Assays, Ann Arbor, MI, USA). Cystatin C level was measured using a cystatin C Elisa kit (W Systems, Minneapolis, MN, USA). LPO level was measured by reacting with thiobarbituric acid (TBA) as described previously [33].

### Measurement of urine parameters

Before mice were sacrificed, urine samples were collected in a metabolic cage for 24 h and centrifuged at 3,000 rpm for 10 min. Albumin excretion was determined with competitive ELISA (ALPCO, Westlake, OH, USA). Urine creatinine level was measured by a modified Jaffe method and creatinine clearance was calculated using a standard formula.

KIM1 was analyzed using mouse specific KIM1 ELISA assay kit purchased from R&D Systems (Minneapolis, MN, USA). Urine aliquots were used in duplicate in each assay following the manufacturer’s recommended protocols.

### Measurement of kidney LPO

LPO in kidney tissue was measured with the LPO assay kit (Cayman Chemical Co, Ann Arbor, MI, USA) according to the manufacturer’s recommended protocols.

### Histology and immunohistochemistry

For each mouse, quantitative analysis of glomerular volume, fractional mesangial area, and tuft area in paraffin-embedded kidney sections stained with PAS reagent was performed as described previously [33]. To examine collagen matrix, paraffin-embedded sections were stained with a picrosirius red stain [33]. Oil Red O staining was performed to evaluate lipid accumulation in frozen kidney tissues as described previously [33]. For immunohistochemistry, we used anti-nephrin (1:100; Progen biotechnik GmbH, Heidelberg, Germany), anti-F4/80 (1:200; Santa Cruz Biotechnology, Inc., Santa Cruz, CA, USA), anti-αSMA (1:200), and anti-collagen IV (1:200; Southern Biotechnology Associates, Birmingham, AL, USA) antibodies. Images were captured using a Zeiss microscope equipped with an Axio Cam HRC digital camera and Axio Cam software (Carl Zeiss, Thornwood, NY, USA). Staining intensities were then quantified using Image-Pro Plus 4.5 software (Media 149 Cybernetics, Silver Springs, MD, USA) as described previously [33]. Imaging for DAPI (1:1000), anti-PMP70 (1:200, Abcam, Cambridge, MA), and anti-catalase (1:200, Santa Cruz Biotechnology) antibodies was conducted using a confocal microscope (Carl Zeiss, Gottingen, Germany).

### Measurement of superoxide generation

Kidney tissues from control or STZ-induced diabetic mice treated with or without APX-115 or losartan, were embedded as frozen block. DHE (5 µM, Molecular Probes) was applied to the freshly cut frozen kidney segments (10 µm) for 10 min at 37°C to reveal the presence of ROS with red fluorescence at 561 nm followed by DAPI staining and imaging by LSM880 with airy scan (Carl Zeiss Microscopy GmbH, 07745 Jena, Germany). Fluorescence intensities were then quantified using Image-Pro Plus 4.5 software (Media 149 Cybernetics, Silver Springs, MD, USA).

### Real-time RT-PCR analysis

Total RNA was extracted using Trizol reagent (Life Technologies), and mRNA expression was measured by means of real-time PCR performed using an ABI7300 system (Applied Biosystems, Carlsbad, CA, USA) and 20 μL reaction volumes containing cDNA transcripts, primer pairs, and SYBR Green PCR Master Mix (Applied Biosystems) as described previously [46]. Primer sequences are shown in Table 1.

### Western blot analysis

Kidney tissues were lysed and centrifuged at 13,000 rpm at 4°C for 15 min. The concentration of protein was determined using the Bradford methods (Bio-Rad Laboratories, Hercules, CA, USA), and aliquots of tissue homogenates were mixed with sample buffer containing SDS and β-mercaptoethanol and heated at 95°C for 5 min. The samples were then applied to a SDS-PAGE gel and separated by electrophoresis. The proteins were transferred onto a polyvinylidene fluoride membrane (GE Healthcare BioSciences Co., Piscataway, NJ, USA). The membrane was blocked for 1 h at room temperature with 5% skim milk in TBS-Tween 20 buffer. Catalase, PEX5, PMP70, ACOX1, and β-actin proteins were measured by standard western blot analysis as described previously [46] using anti-catalase (1:3000, Santa Cruz Biotechnology), anti-PEX5 (1:3000, Cell Signaling Technology), anti-PMP70 (1:2000, Abcam), anti-ACOX1 (1:1000, Santa-Cruz Biotechnology), anti-PGC1α (1:2000, Santa-Cruz Biotechnology), and anti-β-actin (1:3000, Sigma-Aldrich) antibodies followed by an overnight incubation at 4°C. After washing, the membranes were developed with an enhanced chemiluminescence detection reagent (GE Healthcare BioSciences Co.) according to the manufacturer’s instructions. Positive immunoreactive bands were quantified using a densitometer (LAS-3000, FUJIFILM Corporation, Tokyo, Japan), normalized by β-actin, and compared to each control.

### Cell culture

A murine mesangial cell line (MES-13, cloned from mice transgenic for the early region of SV-40 virus, passage 25) was obtained from American Type Culture Collection (ATCC, Rockville, MD, USA). Dulbecco’s modified Eagle’s medium (DMEM, Gibco, Life Technologies) containing 5.6 mM glucose was used to cell culture, unless otherwise stated. Cells were maintained at 37°C under 5% CO_2_ in humidified condition in DMEM containing 5% fetal bovine serum (FBS; PAN BIOTECH), and 1% antibiotic-antimycotic solution (Welgene, Daegu, Korea LS 203-01). Cells were plated onto 35 mm culture dishes and near-confluent cells were incubated with serum-free media for 3 h to arrest and synchronize the cell growth.

### Measurement of intracellular ROS by DCF-DA

After serum starvation, mesangial cells were incubated either 5.5 mM glucose or 30 mM glucose for 24 h and then angII (1 µM, Sigma, St. Louis, MO, USA) for 30 min. To see the effect of APX-115, 1 µM APX-115 was treated for 30 min before treating high glucose. The cells were then washed with Hanks’ balanced salt solution (HBSS) and incubated for 10 min in the dark at 37°C HBSS containing 10 µM DCF-DA (Molecular probes). Fluorescence of oxidized DCF was detected using Zeiss LSM880 airy scan (Carl Zeiss Microscopy GmbH, 07745 Jena, Germany) at excitation wavelengths of 488 nm. Four fields of each cell were randomly selected and the fluorescence intensity was measured with a Zeiss vision system (LSM880 with airy scan) and the mean relative fluorescence intensity was measured by the average of random four values.

### Statistical analysis

All results are expressed as mean ± standard error (SE). Statistical significance of differences among groups were compared by analysis of variance followed by Fisher post-hoc analysis. Statistical significance was considered when *p* value was less than 0.05.

## SUPPLEMENTARY MATERIALS FIGURE


