# Supplementary material for: A novel pan-Nox inhibitor, APX-115, protects kidney injury in streptozotocin-induced diabetic mice: possible role of peroxisomal and mitochondrial biogenesis
Source: Oncotarget. 2017 Jun 16;8(43):74217–32. doi: 10.18632/oncotarget.18540 (PMC5650335; doi:10.18632/oncotarget.18540)
Supplement: Supplementary file 1 [file oncotarget-08-74217-s001.pdf]

# A novel pan-Nox inhibitor, APX-115, protects kidney injury in streptozotocin-induced diabetic mice: possible role of peroxisomal and mitochondrial biogenesis

## SUPPLEMENTARY MATERIALS

## MATERIALS AND METHODS

### Immunohistochemistry of 8-oxo-dG and nitrotyrosine

Paraffin-embedded kidney sections were stained anti-8-oxo-dG (1:400; Trevigen Inc., Trevigen, USA) and anti-nitrotyrosine (1:200; Santa-Cruz Biotechnology) antibodies. Original magnification: 100×; scale bar: 100 μm. Images were captured using a Zeiss microscope equipped with an Axio Cam HRC digital camera and Axio Cam software (Carl Zeiss, Thornwood, NY, USA). Staining intensities were then quantified using Image-Pro Plus 4.5 software (Media 149 Cybernetics, Silver Springs, MD, USA).

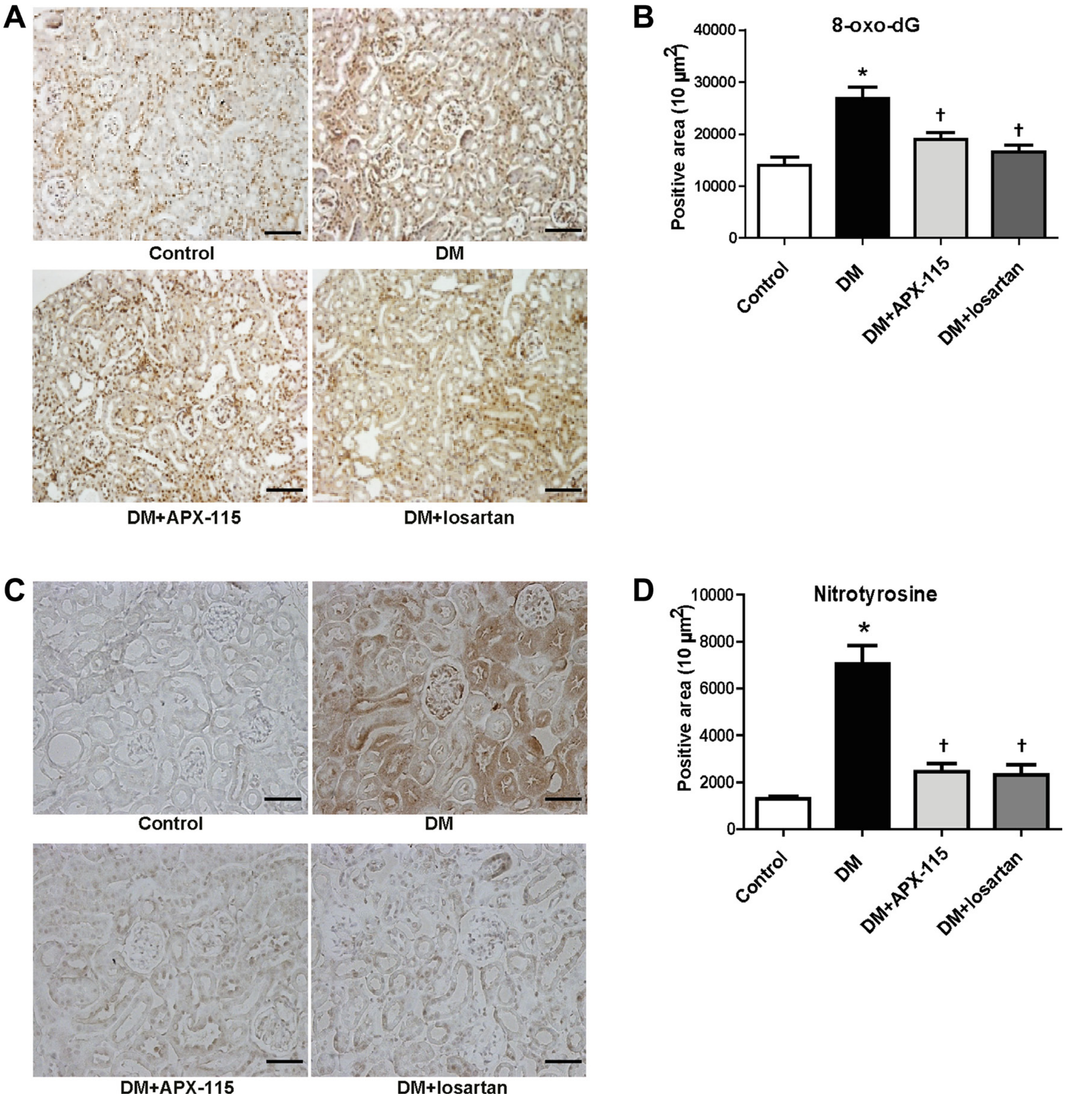


**Supplementary Figure 1: Effect of APX-115 on oxidative stress in the kidneys.** Paraffin-embedded kidney sections were stained with (**A** and **B**) anti-8-oxo-dG (1:400) and (**C** and **D**) anti-nitrotyrosine (1:200) antibodies. Original magnification: 100×; scale bar: 100 µm. Data are presented as means ± SE of 10–12 mice/group; *$p < 0.05$ vs. control, $^{\dagger}p < 0.05$ vs. DM.
